# Complete Inversion of the Uterus of Nearly Twelve Years’ Standing

**Published:** 1858-09

**Authors:** 


### *Complete Inversion of the Uterus of nearly Twelve Years' Duration.*
By Dr. Tyler Smith.

Dr. Tyler Smith read before the Royal Medical and Chirurgical Society ' April 13th, 1858, a case of this.

The author commenced his paper by referring to two cases of inversion of the uterus, published in the Medico-Chirurgical Transactions, in one of which extirpation was practised with recovery of the patient, while in the other, treated by palliative measures alone, death ensued eighteen months after delivery. He brought forward the present case as illustrating a new principle of treatment. Hitherto the cases in which reïnversion has been accomplished have been chiefly limited to cases of recent origin. It has been held that unless the inversion could be reduced soon after the accident, there was little hope of accomplishing it, death generally occurring at periods varying from a few months to five or six years. The operation of extirpating the uterus by ligature is a very serious one. Of thirty-four cases of extirpation, twenty-seven recovered and seven died; in nine of these cases the inverted uterus was mistaken for polypus. The subject of the present case was delivered at the age of eighteen, of a first child, and inversion occurred at that time, but was not suspected by her attendant. When at length an examination was made, a tumor was found in the vagina, but the opinion of those who saw the case was divided between polypus and inversion. Flooding continued to a greater or less extent for nearly twelve years, during which time she was never for a single day free from sanguineous discharge. All attempts at replacing the uterus by those who considered it a case of inversion failed. The patient was sent to the author of the paper in September, 1856, by Mr. Griffith, of Port Madoc, North Wales, under whose care she had been for a short time. The symptoms of anæmia existed in the most marked degree. She was subject to epileptiform convulsions, and frequent faintings. The drain of blood seemed to replace the other secretions to a considerable extent. She passed very little urine, and frequently went twenty-four hours without micturition. On examination, the uterus was found to be completely inverted, the neck of the uterus and the os uteri being very small and rigid. The author determined to attempt its reduction by continuous pressure, with the intention of dilating or developing the os and cervix uteri. With this object the right hand was passed into the vagina night and morning, and the uterus squeezed and moulded for about ten minutes at a time. Chloroform, which had been found so useful in cases of inversion of shorter standing, was not used, because of the feeble state of the heart and circulation, and the comparative absence of pain. In the intervals between these manipulations, in which the author was assisted by Dr. Vernon, the vagina was distended, and firm pressure exerted upwards by a large air pessary. These means gradually dilated the os uteri to such an extent as to allow of the partial return of the uterus, and on the eighth day from the commencement, complete re-inversion took place. The subsequent recovery of the patient was perfect. She has since menstruated regularly, and is in excellent health. The author combats the prevailing notion as to the immobility and unyielding condition of the os uteri in long standing cases of inversion, alluding to the readiness with which the uterus increases, diminishes, and alters in size, under appropriate stimuli. No amount of force will suddenly reduce a case of chronic inversion, but he believes that by air or fluid pressure, so as to convert the fundus and body of the uterus into a wedge, the os uteri may be slowly enlarged in any case so as to admit of reïnversion. Since the presentation of the paper, the author has been informed that the patient is now in the fifth month of pregnancy. The paper concluded by a reference to other conditions, in which air or fluid pressure had been of service, such as the arrest of flooding in abortion, placenta prævia, the expansion of the pelvis in cases of high deformity from mollities ossium, and the induction of premature labor.

The President said the author had observed in his paper that, owing to the condition of the patient, he did not think it prudent to use chloroform. He was evidently aware that cases had been recently recorded in which inversion of the uterus of long standing, though not so long as in the present instance, had been completely cured by pressure with the hand, under the influence of chloroform. He wished to ask the opinion of the author as to whether the use of chloroform would not cause the pressure to be effected in a much shorter space of time, with much less distress to the patient, and perhaps, even with more safety.

Dr. Tyler Smith said his reason for not using chlorcform was, the extremely feeble state of the circulation. He should have been afraid to keep the patient under the influence of chloroform tor the time necessary to manipulate the uterus. Besides, the os uteri was so small that it would have been impossible to have done it at one or even at several sittings. At first, he could make no impression whatever, and he believed he could not have returned the uterus by pressure with the hand alone. It was only after the continuous use of the air pessary that he found the tumor receded at all. The reason why the pessary was forced through the os uteri, he believed was, that by the influence of the pressure, the os uteri was developed. It was by the process of development, rather than by the operative pressure, that the uterus was reïnverted. Then, again, the operation not being painful, there was no bar to the use of considerable efforts to reïnvert the uterus. The process of reïnversion had often been tried in her case.

The President: With chloroform?

Dr. Tyler Smith: Without chloroform. The use of chloroform, he conceived. would be in permitting the relaxation of the os uteri; but in this case the os uteri was so small, that dilatation at any one sitting, would not, he believed, have effected the object.

Dr. Snow did not think that the slow state of the circulation need have been any bar to the administration of chloroform for ten minutes or so during manipulation. He had given chloroform to several patients, in operations for hæmorrhoids, who were reduced to the lowest state from previous hæmorrhage, and anæminated to the greatest degree, and he never saw any ill effects from the chloroform in those cases.

Dr. T. Smith asked Dr. Snow if he would use chloroform for a patient subject to repeated faintings. He once saw chloroform used for the extraction of teeth in the case of a lady who had lost a very large quantity of blood by abortion, and he certainly feared she would die. From what he had seen in that and in other cases, he should fear the use of chloroform for a patient who had lost blood to such an extent as to be frequently subject to fainting. The patient whose case he had described scarcely passed a day without fainting.

Dr. Snow believed that a patient who was liable to fainting, would get through an operation better with chloroform than without it; but of course there was a limit to what might be done either with or without chloroform, when the patient was in an extreme degree of faintness.

Dr. Mackenzie said the author had described the case as one of complete inversion, but it appeared to him (Dr. Mackenzie) that it scarcely came within that category. The late Dr. Hamilton, of Edinburgh, published a case of complete inversion, in which, upon simple treatment, the patient was enabled to live fourteen years with little or no inconvenience. The distinction he laid down between partial and complete inversion was, that partial inversion was attended with hæmorrhage, while complete inversion was not necessarily so attended. The history of the case, as detailed by Dr. Smith, brought it within the category of cases that he (Dr. Mackenzie) had seen, in which the inversion was partial, in which the body or cervix of the uteri was constricted by the os, and in which hæmorrhage necessarily occurred. He had lately met with a case of inversion that ended fatally. He was not at the time aware that inversion or reposition had been effected after a lengthened period; but he found on consulting various journals that from periods averaging from three months to eighteen months or two years, reposition had been effected under chloroform without difficulty by mere manipulation.—*Medical Times and Gazette*, from *Am. Jour. Med. Sciences.*

